# microRNA172 plays a crucial role in wheat spike morphogenesis and grain threshability

**DOI:** 10.1242/dev.146399

**Published:** 2017-06-01

**Authors:** Juan Manuel Debernardi, Huiqiong Lin, George Chuck, Justin D. Faris, Jorge Dubcovsky

**Affiliations:** 1 Department of Plant Sciences, University of California, Davis, CA 95616, USA; 2 Plant Gene Expression Center, University of California, Berkeley, Albany, CA 94710, USA; 3 USDA–ARS Cereal Crops Research Unit, Northern Crop Science Laboratory, Fargo, ND 58102, USA; 4 Howard Hughes Medical Institute, Chevy Chase, MD 20815, USA

**Keywords:** Wheat, Spike development, miRNA, Flowering, AP2, *Q* gene

## Abstract

Wheat domestication from wild species involved mutations in the *Q* gene. The *q* allele (wild wheats) is associated with elongated spikes and hulled grains, whereas the mutant *Q* allele (domesticated wheats) confers subcompact spikes and free-threshing grains. Previous studies showed that *Q* encodes an AP2-like transcription factor, but the causal polymorphism of the domestication traits remained unclear. Here, we show that the interaction between microRNA172 (miR172) and the *Q* allele is reduced by a single nucleotide polymorphism in the miRNA binding site. Inhibition of miR172 activity by a miRNA target mimic resulted in compact spikes and transition from glumes to florets in apical spikelets. By contrast, overexpression of miR172 was sufficient to induce elongated spikes and non-free-threshing grains, similar to those observed in three *Q* loss-of-function mutations. These lines showed transitions from florets to glumes in the basal spikelets. These localized homeotic changes were associated with opposing miR172/*Q* gradients along the spike. We propose that the selection of a nucleotide change at the miR172 binding site of *Q* contributed to subcompact spikes and free-threshing grains during wheat domestication.

## Abstract (precis)

**Highlighted Article:** A nucleotide change in the microRNA172 binding site of the AP2 gene *Q* played a critical role in wheat domestication and the origin of free-threshing modern wheats. See also Greenwood et al. in this issue.

## INTRODUCTION

Wheat, which was domesticated ∼10,000 years ago, has since spread worldwide and become a major crop. The transition from wild to domesticated emmer involved changes at the *Br* (*brittle rachis*) loci that favored the transition from disarticulating to non-shattering spikes. Additional changes resulted in the loss of tough glumes, converting hulled wheats into free-threshing durum and common wheat (Dubcovsky and Dvorak, 2007).

The primary genetic determinants of the free-threshing habit are recessive mutations at the *Tg* (*tenacious glume*) gene and a hypermorphic mutation at the *Q* gene (Faris et al., 2014). *Q* also controls the subcompact spike phenotype and influences other domestication-related traits such as rachis fragility, glume toughness, plant height and flowering time (Faris et al., 2005). The *Q* allele is present in most cultivated wheats, whereas wild and cultivated emmer have the *q* allele, which is associated with a speltoid spike (a spear-shaped spike with an elongated rachis) and non-free-threshing grains (Faris et al., 2005). However, high doses of the *q* allele can also render compact spikes and free-threshing grains (Muramatsu, 1963).

**Fig. 1.**
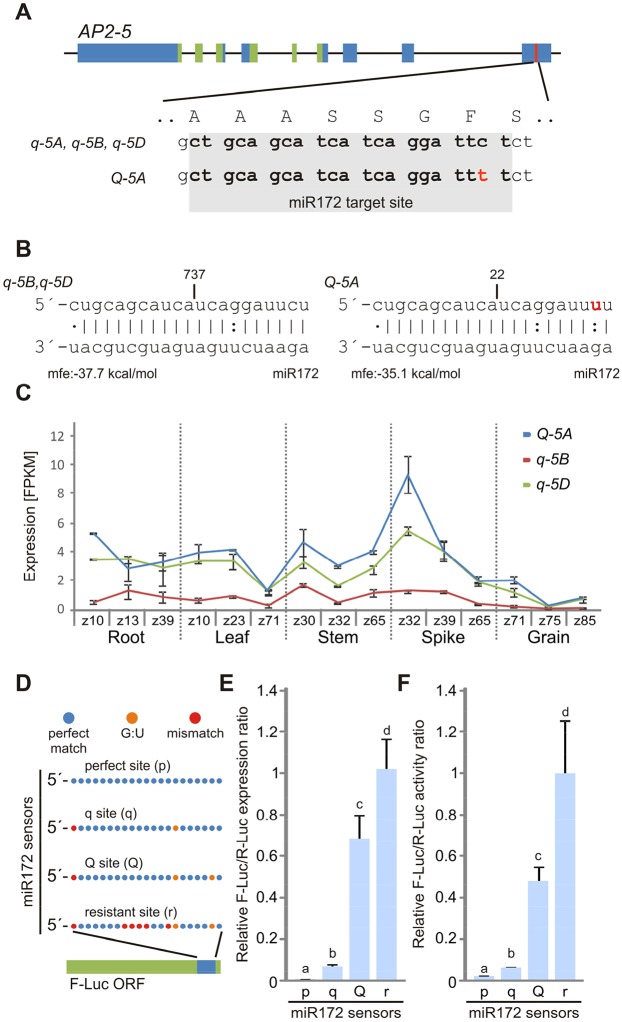
**The *Q* allele has a weaker miR172 target site than the *q* allele.** (A) Genomic structure of the wheat *AP2-5* gene and sequence variation at the miR172 target site between *Q* and *q* alleles in different homoeologs. The regions encoding the AP2 domains are indicated in green and the miR172 target site is in red. The *q* alleles in the three wheat homoeologs harbor identical miR172 target sites. The single nucleotide polymorphism in the *Q-5A* allele is indicated in red. (B) Schematic representation of the interaction between miR172 and *q*/*Q* target sites. The 5′ ends of the degradome products are indicated at the top by a vertical line along with the number of detected reads. The predicted energy of the interactions is indicated beneath the sequences. mfe, minimum free energy (see the supplementary Materials and Methods). (C) Expression of *AP2-5* homoeologs in different tissues and developmental stages from hexaploid wheat. z, Zadoks scale (Zadoks et al., 1974). (D) Schematic of firefly luciferase sensors (F-Luc) with different miR172 target sites, with red indicating a mismatch, orange a G:U interaction and blue a perfect match with miR172 within the target site. (E,F) *N. benthamiana* leaves were co-infiltrated with F-Luc sensors and a vector overexpressing miR172. *Renilla* luciferase (R-Luc) was used as an internal control. Expression was first normalized to R-Luc and then adjusted using the miR172-resistant sensor. (E) Relative transcript levels of each F-Luc sensor estimated by qRT-PCR. (F) Relative luciferase activity of each F-Luc sensor. Bars represent mean±s.e.m. of six (qPCR) and three (luciferase) replicates, and different letters on top indicate statistically significant differences (*P*<0.05) by non-parametric Student–Newman–Keuls test.

The cloning of the *Q* gene showed that it encodes an AP2-like transcription factor (TF) that carries two plant-specific AP2 domains (Simons et al., 2006). The main *Q/q* polymorphism is on chromosome 5A (*AP2*-*5A*), the 5B homoeolog (*AP2*-*5B*) is a pseudogene, and the 5D homoeolog (*AP2*-*5D*) encodes a functional protein that contributes to the suppression of the speltoid phenotype (Zhang et al., 2011). Like other *AP2-*like genes, *Q* and its homoeologs have a miR172 target site within the coding region close to the 3′ end of the gene (Fig. 1A) that alters mRNA stability. This regulation has been implicated in multiple developmental processes in a wide range of plant species (Aukerman and Sakai, 2003; Chen, 2004; Schwab et al., 2005; Zhu and Helliwell, 2011; Huijser and Schmid, 2011; Houston et al., 2013; Chuck et al., 2007; Nair et al., 2010; Lee and An, 2012).

Analysis of *q* and *Q* allele sequences (henceforth, *q-5A* and *Q*-*5A*, respectively) from several wheat accessions revealed polymorphisms at two positions (Simons et al., 2006; Zhang et al., 2011; Chuck et al., 2007; Sormacheva et al., 2015). The first is a G-to-A transition in exon 8, which generates a non-synonymous change, V329I, after the two AP2 domains (Simons et al., 2006). The second is a synonymous mutation that lies within the miR172 target site in exon 10. The point mutation in the *Q* alleles replaces a strong G-C pair by a G:U wobble in the interaction with miR172, which reduces the predicted energy of the interaction (Fig. 1B). In addition, this weaker G:U interaction is located in the 5′ region of the miRNA, which is crucial for plant miRNA-mediated target repression (Mallory et al., 2004; Liu et al., 2014; Schwab et al., 2005).

In this study, we show that the mutation in the miR172 target site in the *Q* allele results in less effective targeting by the miRNA. Furthermore, using transgenic wheats that express an artificial miR172 target mimic and miR172 overexpression constructs, we show that perturbations in the balance between the miRNA and *Q* have a profound effect on spike development and contribute to the free-threshing character. Finally, we show a gradient of homeotic transformations along the vertical axis of the transgenic spikes that is associated with opposing expression gradients of miR172 and *Q* in the developing spike.

## RESULTS

### miR172 cleaves *Q* and *q* transcripts with different efficiency

Using published RNA-seq data and the WheatExp web tool (Choulet et al., 2014; Pearce et al., 2015), we determined that the three *AP2-5* homoeologs have similar expression patterns across tissues and developmental stages. However, the *Q-5A* homoeolog shows the highest transcript levels in young spikes (∼3-5 mm in length) at Zadoks stage 32, when the elongating stem has two nodes (Zadoks et al., 1974) (Fig. 1C). This is not surprising since the *Q-5A* allele is expressed at higher levels than *q-5A* (Simons et al., 2006), and the B and D genome homoeologs (henceforth, *q-5B* and *q-5D*, respectively) carry the *q* allele at the miR172 target site (Fig. 1A).

We then used published genome-wide degradome data (Addo-Quaye et al., 2008; German et al., 2008) for hexaploid wheat (Sun et al., 2014; Tang et al., 2012) to test if the differences in transcript levels correlate with differences in mRNA cleavage by miR172 (see the supplementary Materials and Methods). The miR172 target site in *Q-5A* has three sites of non-perfect complementarity, compared with two in the *q-5B* and *q-5D* homoeologs (Fig. 1B), so we expected it to be cleaved with lower efficiency. The *q-5B* and *q-5D* cleavage products are undistinguishable so we counted them together. The reads carrying the expected miR172-mediated degradation product for the combined *q-5B* and *q-5D* alleles were more than 36-fold (737 reads) higher than those from the *Q-5A* homoeolog (22 reads, Fig. 1B). Because transcript levels are similar or higher for *Q-5A* than for *q-5B* and *q-5D*, the lower abundance of the *Q-5A* cleavage products suggests a reduced miR172 cleavage efficiency for *Q* relative to the *q* homoeologs.

To test this hypothesis, we developed four sensors with different miR172 target sites using a dual-luciferase sensor system (Liu and Axtell, 2015). We inserted the *Q* and *q* miR172 target sites, a target site with perfect complementarity to miR172 (positive control) and a resistant site with five extra mismatches (negative control) into the open reading frame of the firefly luciferase gene (*F-Luc*, see the Materials and Methods) (Fig. 1D). Co-infiltration of each of the sensors with miR172 into *Nicotiana benthamiana* leaves using *Agrobacterium* showed significant differences in transcript levels (Fig. 1E). As expected, the perfectly paired sensor (positive control) exhibited the lowest transcript levels and the resistant site (negative control) the highest (Fig. 1E). Most importantly, the sensor with the *Q* target site showed significantly higher transcript levels than the sensor with the *q* site (Fig. 1E). This confirmed that the *Q* site is less effective than the *q* site as a cleavage target of miR172. In addition, the transcripts of the sensor with the *q* site were higher than the positive control (Fig. 1E), which suggests that the mismatch and the G:U pair in the *q* site have some effect on limiting cleavage efficiency. Conversely, the transcripts of the sensor with the *Q* site were significantly lower than the negative control (Fig. 1E), which suggests that the *Q* site has a residual repression by miR172. The luciferase activity of these samples showed a pattern similar to that observed for the transcript profiles (Fig. 1F), indicating that the transcriptional differences were retained at the protein level.

### Reduced activity of miR172 mimics the phenotype of plants with high expression of *Q*

The above results suggest that the lower efficiency of miR172 to cleave *Q* than *q* transcripts could play a role in the morphological differences previously observed between plants carrying these alleles. In wheat, we identified five members per haploid genome for the miR172 family, which lead to three putative mature miRNA variants (Fig. S1A). Available small RNA-seq data (Fig. S1B) and qRT-PCR analysis (Fig. S1C) indicated that miR172 is expressed mainly in reproductive tissues. This is consistent with a role of this miRNA in reproductive development (Aukerman and Sakai, 2003; Chen, 2004; Zhu and Helliwell, 2011; Huijser and Schmid, 2011; Houston et al., 2013; Chuck et al., 2007; Nair et al., 2010; Lee and An, 2012), and with the higher expression of *Q-5A* relative to the other homoeologs in young spikes (Fig. 1C).

**Fig. 2.**
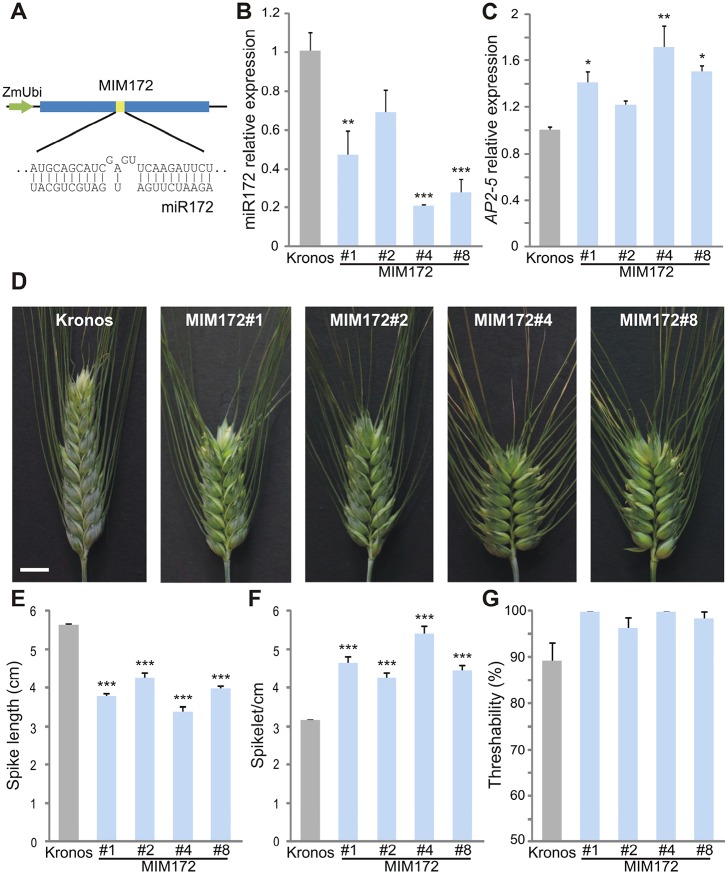
**Downregulation of miR172 results in compact spikes.** (A) The MIM172 vector, showing the miR172 complementary site including the extra bases that preclude cleavage. ZmUbi, maize *UBIQUITIN* promoter. (B,C) Expression level of mature miR172 (B) and *AP2-5* combined homoeologs (C) estimated by qRT-PCR in four independent MIM172 transgenic lines. Expression levels were normalized to the Kronos control (*n*=4). (D) Primary spike of Kronos and the different MIM172 transgenic plants 3 weeks after heading. Scale bar: 1 cm. (E,F) Spike length (from the base of the first and last spikelets) (E) and spikelets per cm (F) in the primary spike of Kronos and the different transgenic plants 3 weeks after heading (*n*=10). (G) Threshability (grains threshed in one pass through an electric thresher/total number of grains, *n*=8). No statistical analysis is presented for the threshability data because values were 100% for all plants in two MIM172 transgenic lines. All bars represent mean±s.e.m. and asterisks indicate a statistically significant difference to the wild-type control (*P<0.05, **P<0.01, ***P<0.001) by Dunnett's test.

We used the artificial target mimicry approach (MIM) (Franco-Zorrilla et al., 2007) to reduce the activity of miR172 *in vivo* and test its role in spike development. We generated four independent transgenic Kronos plants (*Q-5A* genotype) expressing a MIM172 construct driven by the maize *UBIQUITIN* promoter (Fig. 2A). All MIM172 transgenic plants had lower levels of miR172 than the wild-type control, which were accompanied by higher transcript levels of the *AP2*-*5* homoeologs (Fig. 2B,C). The differences were significant in three out of the four tested transgenic events.

Under the conditions tested in this study, the spikes of MIM172 were significantly shorter than those of Kronos (Fig. 2D,E) despite carrying the same number of spikelets (Fig. S2A), which resulted in more-compact spikes (more spikelets/cm, Fig. 2F). The transgenic line with the lowest level of miR172 (MIM172#4) and highest level of *AP2-5* homoeologs showed the most compact spike (75% more compact than Kronos, Fig. 2F).

In addition to the changes in spike compactness, the MIM172 spikes were easier to thresh (Fig. 2G), the plants were shorter, and the strongest lines MIM172#4 and #8 flowered a few days later than non-transgenic controls (Fig. S2B,D,E). These results are similar to those described for transgenic wheat plants overexpressing *Q* (Simons et al., 2006) or with increased *Q* copy number (Muramatsu, 1963). No differences were observed between Kronos and MIM172 in the lodicules, which are known to be affected by point mutations in the miR172 site of a paralogous *AP2-2* in barley chromosome 2H (Houston et al., 2013; Nair et al., 2010) (Fig. S2F). Even though the maize *UBIQUITIN* promoter used in this study is expressed in the lodicules of transgenic wheat plants (Rooke et al., 2000), we cannot rule out the possibility that a stronger MIM172 or higher levels of *Q* could have an effect on the lodicules.

We then crossed the MIM172#4 line with a tetraploid *T. turgidum* subsp. *dicoccon* accession CItr 14454 (*T. dic* 454) homozygous for the wild-type *q-5A* allele. F_1_ *Qq* plants carrying the MIM172 transgene had lower levels of miR172 and increased transcript levels of both *Q-5A* and *q-5A* alleles than F_1_ plants without the transgene (Fig. S3A-C). This indicates that miR172 is effective at modulating the transcript levels of both alleles. The spikes of *Qq* plants without the MIM172 transgene were speltoid and had tough glumes (Fig. S3D-G), an expected result since the *Q-5A* allele is hypermorphic but not dominant. The spikes of *Qq* plants carrying the MIM172 transgene were compact and the glumes were softer with a less pronounced keel (Fig. S3D-H), similar to homozygous *Q* plants transgenic for MIM172.

Overall, these results show that miR172 plays an important role in wheat spike development, and a reduced activity of miR172 favors domesticated traits such as a more compact spike and free-threshing grains.

### Higher levels of miR172 promote wild traits in spikes

**Fig. 3.**
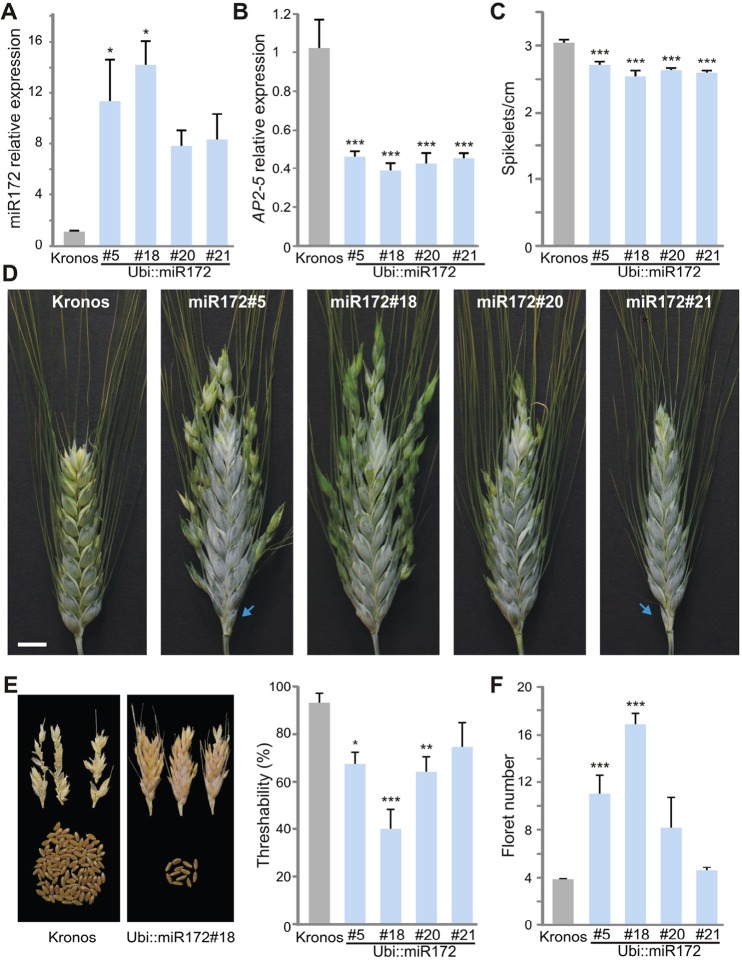
**Overexpression of miR172 promotes wild traits in spikes and an increased number of florets.** (A,B) Expression levels estimated by qRT-PCR of mature miR172 (A) and *AP2-5* combined homoeologs (B). Expression levels were normalized to Kronos (*n*=4). (C) Spikelets per cm in the primary spike of Kronos and the different transgenic plants 3 weeks after heading (*n*=10). (D) Primary spikes of wild-type Kronos and four independent Ubi::miR172 transgenic lines showing spikelets with higher numbers of florets (3 weeks after heading). Arrows indicate rudimentary spikelets at the base of the spikes. Scale bar: 1 cm. (E) (Left) Threshed spikes from Kronos and Ubi::miR172#18 line. (Right) Threshability (*n*=8). (F) Number of florets per spikelets in the primary spike (*n*=10). All bars represent mean±s.e.m. and asterisks indicate statistically significant difference to the wild-type control (*P<0.05, **P<0.01, ***P<0.001) by Dunnett's test.

We then tested the effect of increased activity of miR172 on spike development by overexpressing the *miR172d* gene from chromosome arm 6AL under the control of the maize *UBIQUITIN* promoter (Ubi::miR172). We obtained four independent Kronos transgenic lines with higher levels of miR172 (Fig. 3A) and reduced levels of the *AP2-5* homoeologs (Fig. 3B).

The spikes of the Ubi::miR172 transgenic lines were speltoid in shape and had significantly longer spike internodes than the wild-type Kronos (Fig. 3C,D). These plants showed a reduced number of spikelets without changes in spike length (Fig. S4A,B). In addition, the transgenic lines were taller than the control Kronos (Fig. S4D,E) and flowered a few days earlier (Fig. S4C). The glumes of the Ubi::miR172 lines were firm and adhered strongly to the grains, and showed a reduced angle (Fig. S5) resulting in spikes that were more difficult to thresh (Fig. 3E). These results indicate that high levels of miR172 can modify spike architecture and reduce threshability, even when the *Q* allele with the V329I amino acid change is still present.

We also analyzed the florets of the central spikelets of the Ubi::miR172 plants and found normal lodicules, stamens and carpels (Fig. S6A-C). Interestingly, the spikelets of Ubi::miR172 transgenic plants have significantly more florets (Fig. 3D,F, Fig. S6B). For example, in transgenic lines #5 and #18 the average number of florets was ∼3- to 4-fold higher than that of the wild-type Kronos control. We then re-analyzed the MIM172 plants with reduced miR172, and observed the opposite effect: the number of florets per spikelet was slightly reduced in three of the four transgenic lines. However, the differences were significant only in line #4 (Fig. S2C), which also exhibited the lowest levels of miR172 and most compact spikes (Fig. 2B,D). These results indicate that the levels of miR172 also affect the number of floret meristems produced during spikelet development.

### Truncation mutations in *Q-5A* result in non-free-threshing spikes with more florets per spikelet

**Fig. 4.**
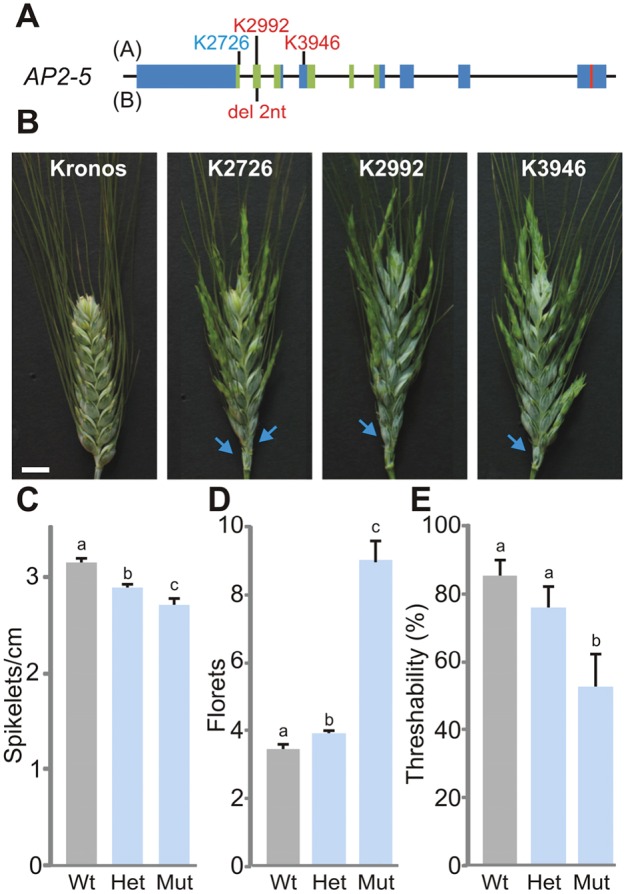
***Q-5A* mutations affect spikelet density, number of florets per spikelet and threshability.** (A) Genomic structure of the wheat *AP2-5* gene showing the position of the Kronos mutations K2726, K2992 and K3946 in the A homoeolog (top) and the 2 nt deletion in the B homoeolog (beneath). (B) Primary spike of Kronos and three *Q-5A* mutants 3 weeks after heading. Arrows indicate rudimentary spikelets at the base of the spikes. Scale bar: 1 cm. (C-E) Progeny of the cross K3946×Kronos genotyped and classified as homozygous wild type (Wt), homozygous mutant (Mut) and heterozygous (Het). (C) Spikelets per cm in the primary spike. (D) Number of florets per spikelet in the primary spike. (E) Threshability (*n*=9). Different letters above the bars indicate statistically significant differences (*P*<0.05) by Student–Newman–Keuls test.

To test if the increased number of florets in Ubi::miR172 plants was associated with *AP2-5* or other targets, we obtained three independent *Q-5A* mutants in a TILLING population generated in Kronos (Uauy et al., 2009; Krasileva et al., 2017). We identified a total of 84 mutations in the coding region of *Q-5A* and selected two that generated premature stop codons in the second (K2992) and fourth (K3946) exons and one that eliminated the splicing donor site of the first intron (K2726) (Fig. 4A). It was not necessary to select mutations for the *q-5B* homoeolog because Kronos has a natural deletion of two nucleotides in the second exon that generates a frameshift and a premature stop codon (Fig. 4A), also reported in other tetraploid wheat cultivars (Zhang et al., 2011).

All M_4_ plants homozygous for the three selected *Q-5A* truncation mutations showed spikelets with an increased number of florets (Fig. 4B), indicating that a reduced expression of *Q-5A* is likely to have contributed to this phenotype in the Ubi::miR172 plants. To confirm this result, we crossed line K3946 with Kronos and analyzed 60 F_2_ individuals. We observed that the variation for the *Q-5A* K3946 mutation was linked to spike compactness (Fig. 4C), a higher number of florets per spikelet (Fig. 4D), reduced threshability (Fig. 4E), and an acceleration of a few days in heading time (Fig. S7A).

We observed a trend to taller plants with a decrease in the dosage of the functional *Q-5A* allele, although the differences were not significant (Fig. S7B). A similar increase in height was significant in a non-functional *Q-5A* mutant in hexaploid wheat in the companion study (Greenwood et al., 2017). The magnitude of these height changes is consistent with the difference observed in the Ubi::miR172 transgenic wheat plants (Fig. S4E). For the traits affected in the *Q-5A* truncation mutants, the heterozygous plants displayed an intermediate phenotype (Fig. 4C,E), supporting a dosage-dependent effect of *Q* on these traits. Finally, no differences in mature miR172 levels in the spikes were observed between wild-type and *Q-5A* mutant plants (Fig. S7C), suggesting that a feedback of AP2-5A on miR172 is probably not involved in the mutant phenotypes.

Taken together, our results indicate that a balance between miR172 and *AP2-5* transcript levels is important for normal spike development. Modifications in this balance affect the final spike compactness and shape, the number of florets produced per spikelet, and the free-threshing character.

### Large changes in miR172 and *Q* transcript levels are associated with homeotic changes in glumes and lemmas

**Fig. 5.**
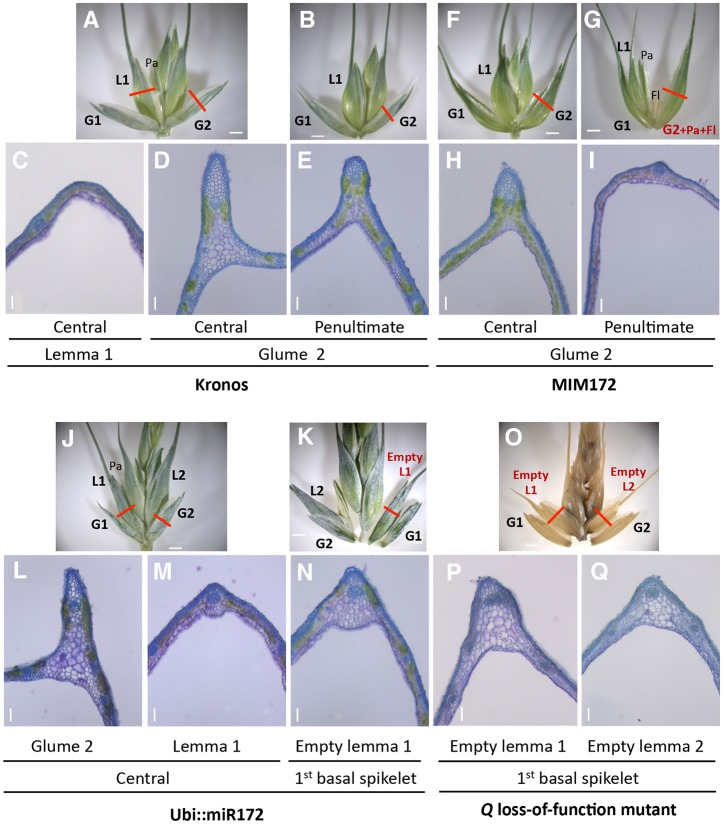
**Transitions between** **glumes and lemmas.** (A-E) Morphological and anatomical traits of Kronos wild type. (A,C,D) Central spikelet with transverse section of the first lemma (L1, C) and second glume (G2, D). (B,E) Penultimate spikelet showing a transverse section of G2 (E; note the reduced keel compared with the central spikelet). (F-I) MIM172#4. (F) Central spikelet showing longer awns and reduced keel in G2 transverse section (H) relative to Kronos. (G) Penultimate spikelet showing a G2 with a palea, floral organs and an anatomy in transverse section (I) similar to L1 in Kronos. (J-N) Ubi::miR172#18. (J) Central spikelet with complete florets 1 and 2. Transverse sections show a pronounced keel in G2 (L) and an incipient keel in L1 (M). (K) Basal spikelet with an empty L1 (no palea or floral organs). (N) Transverse section of L1 showing a more developed keel than the wild type. (O-Q) Loss-of-function mutation in *Q-5A* (K3946). (O) Basal spikelet showing empty L1 and L2 with short awns. (P) Transverse sections of an empty L1. (Q) Transverse sections of empty L2. Note the more developed keel in L1 relative to L2. Pa, palea; Fl, floral organ. Scale bars: 2 mm in spike images; 100 µm in anatomical sections.

Altered balances of miR172 and *AP2-5* were also associated with changes in glumes and lemmas in the basal and apical regions of the spike. To document these changes, we characterized the morphology and anatomy of glumes and lemmas along the spike vertical axis in genotypes with different levels of *Q* expression. In the central spikelets of wild-type Kronos, glumes differed from lemmas by their stronger keels (Fig. 5A-E, Fig. S8A, Fig. S9A), more-abundant sclerenchyma (Fig. S8C, Fig. S9C) and shorter awns (Fig. S10A,B). Interestingly, in the Kronos penultimate spikelet, the length of the glume awns increased slightly (Fig. 5B, Fig. S10A) and the keels were less pronounced (Fig. 5E).

Compared with Kronos, the glumes of the central spikelets of the MIM172#4 transgenic plants showed longer awns (Fig. 5F, Fig. S10A), increased angle (Fig. S5A,B) and reduced keels, which was confirmed in transverse sections of the glumes (Fig. 5H, Fig. S8A,B). These changes were correlated with softer glumes and higher grain threshability in MIM172 transgenic plants relative to Kronos (Fig. 2G). As in Kronos, the glumes of the more apical spikelets showed longer awns (Fig. 5G, Fig. S10A) and even less pronounced keels that made them look more similar to lemmas (Fig. 5I).

A homeotic transition from glumes to lemmas was evident in the more apical spikelets, where the glumes transition gradually to florets with fertile floral organs and grains closer to the terminal spikelet (Fig. 5G). These changes were more frequent and more pronounced in the second glume (G2) than in the first glume (G1) (Fig. S10C). The percentage of G2 showing any of these homeotic changes increased from 10% in the fourth spikelet below the terminal spikelet to 100% in the penultimate spikelet, followed by a decrease to 67% in the terminal spikelet. Homeotic changes in G1 were detected in only 29% of the penultimate spikelets and 22% of the terminal spikelets. We did not observe these changes in the basal and central spikelets of MIM172#4 plants. Similar homeotic changes were first observed in glumes of the hexaploid wheat mutant *Q′* (Greenwood et al., 2017), which has an additional mutation in the miR172 binding site.

The transgenic wheat plants overexpressing miR172 (Ubi::miR172#18; Fig. 5J-N) and the loss-of-function mutants for *Q-5A* (K3946; Fig. 5O-Q) showed the opposite changes. Relative to Kronos, Ubi::miR172 and the *Q-5A* mutants exhibit increased sclerenchyma in glumes and lemmas (Fig. S8C, Fig. S9C), reduced glume angle (Fig. S5A,B) and a more pronounced keel in lemmas (Fig. 5M, Fig. S9A,B). These changes correlate well with their tougher glumes and lemmas, and reduced grain threshability (Fig. 3E, Fig. 4E). Glume awns of these lines were as short as in Kronos but did not show the increased length observed in the apical spikelets of Kronos (Fig. S10A). Both the Ubi::miR172 and *Q-5A* loss-of-function mutants showed one to three rudimentary spikelets in the most basal nodes of the spike (arrows in Figs 3 and 4) and empty lemmas in the fully formed basal spikelets. In Ubi::miR172, the first lemmas of the four basal spikelets frequently showed no subtended palea or floral organs (90% in spikelet 1, 67% in spikelet 2, 30% in spikelet 3, 10% in spikelet 4), whereas the second lemmas had perfect paleas and floral organs in all spikelets. More substantial changes were observed in *Q-5A* loss-of-function mutants, where the first lemmas were empty in all the spikelets of all individuals and 77% of the second lemmas were empty (Fig. 5O). The empty lemmas were more keeled (Fig. 5N,P,Q) than the equivalent lemmas in wild-type Kronos plants (Fig. 5C) and showed shorter awns (Fig. S10B).

**Fig. 6.**
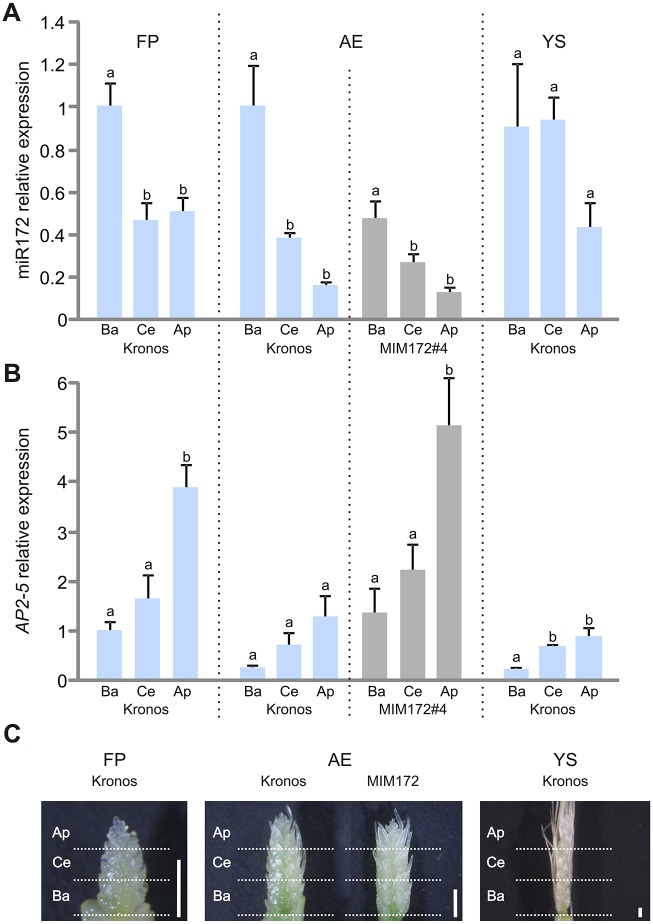
**Spatial distribution of *AP2-5* and miR172 along the vertical axis of developing spikes.** (A,B) Expression levels, as estimated by qRT-PCR, of mature miR172 (A) and *AP2-5* combined homoeologs (B). Expression was analyzed in basal (Ba), central (Ce) and apical (Ap) sections of developing spikes of Kronos at three developmental stages: FP, floret primordia (∼1.5 mm); AE, awn elongation (∼2.5 mm); and YS, young spikes (∼10 mm). Expression was also analyzed in sections of MIM172#4 spikes at AE stage. *AP2-5* transcript and miR172 levels were normalized to the Kronos basal section at FP stage (*n*=3). Different letters above the bars indicate statistically significant differences (*P*<0.05) by Student–Newman–Keuls test. (C) Spikes at the three developmental stages used to study miR172 and *AP2-5* expression. Horizontal dotted lines indicate the three sections analyzed. Scale bars: 1 mm.

We found that the homeotic changes along the spike axis were associated with opposing gradients in *AP2-5* and miR172 expression (Fig. 6). At the three developmental stages tested, miR172 levels were higher at the basal region of the spike and decreased towards the apical region of the spike (Fig. 6A). The opposite trend was observed for *AP2-5*, which showed highest expression levels in the apical part of the spike and lowest in the basal part (Fig. 6B). In addition, we observed that *AP2-5* expression is downregulated with spike development (Fig. 6B), in agreement with the RNA-seq data (Fig. 1C). We observed a similar pattern in the presence of the MIM172 transgene (awn elongation stage, Fig. 6), with higher levels of *AP2-5* transcripts in the apical region and of mature miR172 at the basal region. In MIM172, *AP2-5* reached higher expression levels in all spike sections relative to Kronos. MIM172 plants were the only ones where we detected a faint signal for *AP2-5* in mRNA *in situ* hybridizations in young spikes (Fig. S11). The signal was detected in glume and lemma primordia, which is consistent with the homeotic changes observed in these organs. No signal was observed in *Q-5A* mutants used as negative control (Fig. S11).

## DISCUSSION

### Homeotic changes associated with altered levels of miR172 and *Q*

In the central spikelets, the glumes and lemmas were more similar to each other in MIM172 than in Kronos. This transition was even more evident in the apical spikelets, where glumes morphed into fully formed fertile florets. Greenwood et al. (2017) report similar homeotic changes in a hexaploid line with a mutation in the *Q* miR172 binding site (*Q′*), confirming that the homeotic changes in MIM172 are mediated by *Q*. The glume homeotic changes in *Q′* were found in a higher proportion of spikelets than in our tetraploid MIM172 plants, possibly because of the additional contribution of *q-5D*. The glume homeotic changes in tetraploid wheat were stronger in the second than in the first glume, which is consistent with a gradual shift in homeotic regulation in the boundary between these two adjacent organs.

The hypothesis of a homeotic change is further supported by opposite changes observed in the spikes of Ubi::miR172 and *Q-5A* loss-of-function mutants. In these plants, the first and sometimes the second lemmas were empty (no subtended palea or floral organs), have shorter awns, increased sclerenchyma and incipient keels. The changes were more evident in the basal spikelets, where the first lemma sometimes resembles a third glume. The existence of a gradient in awn length in the *Q-5A* mutant (Fig. S10B) suggests that other genes might redundantly affect this character. These changes were not observed in *Q-5A* loss-of-function mutants in hexaploid wheat (Greenwood et al., 2017), probably because of the presence of a functional *q-5D*.

The homeotic changes in MIM172 were stronger in the apical part of the spike, whereas the changes observed in Ubi::miR172 were stronger in the basal spikelets. In the wild-type Kronos spike, glumes from the apical spikelets have longer awns and less pronounced keels than in the central spikelets. These morphological gradients are consistent with the gradual increase in *Q* transcript levels from the basal to the apical parts of the spike (Fig. 6). A model to explain these localized changes is shown in Fig. S12.

A role of *Q* in homeotic transitions in the external organs of the wheat spikelet is not surprising because in *Arabidopsis* the A-class homeotic gene *AP2* homolog is expressed predominantly in the outer floral whorls, and its loss triggers homeotic changes in sepals and petals (Bowman et al., 1989). Gradual transitions between glumes, empty lemmas and lemmas subtending floral organs have also been described during the evolution and development of grasses (Malcomber et al., 2006). Based on these results, it is tempting to speculate that the higher threshability of MIM172 relative to Kronos (*Q*) and of Kronos relative to tetraploid lines with the *q* allele are the result of different degrees of homeotic transition between glumes and lemmas.

### Regulation of reproductive development by miR172–AP2-like TFs

The miR172 family is highly conserved across plant species and plays multiple roles in plant development. Artificially high levels of miR172 and reduced levels of AP2-like TFs are associated with early flowering and alterations in inflorescence and flower architecture in several plant species (Zhu and Helliwell, 2011). Our transgenic wheat plants overexpressing miR172 (Ubi::miR172) flowered earlier, whereas those with reduced miR172 (MIM172) flowered later than the wild type. These results indicate a conserved role of the miR172–AP2-like system in the regulation of flowering time in wheat.

We did not observe any obvious defects in the stamens, lodicules and carpels of Ubi::miR172 plants, but we did observe an abnormally high number of florets per spikelet. This phenotype was also observed in loss-of-function mutants of *Q-5A* in tetraploid (this study) and hexaploid (Greenwood et al., 2017) wheat, suggesting that the increased number of florets per spikelet in the Ubi::miR172 plants was due, at least in part, to the reduced expression of *Q-5A*. An increase in the number of florets has been associated with a reduced determinacy of the spikelet meristem in other grasses (Lee and An, 2012; Chuck et al., 1998, 2008). In particular, a similar extra floret phenotype was described for maize plants with a null mutation in the *INDETERMINATE SPIKELET 1* (*IDS1*) (Chuck et al., 1998), which encodes an ortholog of AP2-5 (Fig. S1E). These observations indicate a conserved role of *AP2*-like TF genes within the *Q*/*IDS1* clade in limiting the number of florets produced by the spikelet meristems. Under the conditions tested here, the *Q-5A* mutants shared other phenotypes with the Ubi::miR172 transgenic plants, including speltoid spikes and earlier heading time. These results suggest that the effects of miR172 on these traits is likely to be mediated in part by the effect of miR172 on *Q-5A*.

*Q* has also been reported to affect wheat height (Zhang et al., 2011). A small (<10 cm) but significant increase in height was also observed in *Q-5A* mutants by Greenwood et al. (2017). Consistent with these results, we observed a trend towards increasing plant height in the heterozygous and homozygous *Q-5A* mutants (Fig. S7A), but the differences were not significant. We also observed increased plant height in lines overexpressing miR172 (Fig. S4D,E) and reduced plant height in the MIM172 transgenic plants (Fig. S2D,E). Taken together, these results suggest that higher levels of *Q* can contribute to reductions in wheat plant height.

### A single nucleotide polymorphism in the miR172 binding site of the *Q-5A* allele contributed to wheat domestication

miRNA silencing efficiency can be modified by changes in the miRNA sequence (Debernardi et al., 2012) or in its target site sequences (Mallory et al., 2004; Liu et al., 2014). Examples of natural variation in target sites for miRNAs including miR172 have been described in other grass species (Che et al., 2015; Duan et al., 2015; Hu et al., 2015; Jiao et al., 2010; Miura et al., 2010). In maize *Ts6* mutants, single mutations within the miR172 site of the *IDS1* gene cause meristem defects and a lack of pistil abortion in the tassel (Chuck et al., 2007). In cultivated barley varieties, *HvAP2-2L* alleles with impaired miR172 repression result in cleistogamous flowers and in a compact spike phenotype (Houston et al., 2013; Nair et al., 2010). These results suggest that interactions between miR172 and *AP2-*like genes were likely to have been altered multiple times during grass evolution.

Here, we show that a mutation in the miR172 target site of *AP2-5* played a key role in the acquisition of free-threshing character during wheat domestication. Previous studies demonstrated that this trait was associated with the presence of two polymorphisms in the *Q* allele (Faris et al., 2005; Chuck et al., 2007), but the role of these two polymorphisms has remained controversial (Simons et al., 2006; Chuck et al., 2007; Sormacheva et al., 2015). The G-to-A transition in the eighth exon, which results in a V329I amino acid change, has been shown to alter the ability of the Q protein to form homodimers (Faris et al., 2005). However, this potential dimerization has not been demonstrated *in planta* and there are no indications of its role in the determination of the observed phenotype. Even though our study does not rule out a role of this amino acid change in the modulation of some of the domestication traits, our results demonstrate that alteration of miR172 levels alone is sufficient to induce changes in spike compactness and threshability. The non-free-threshing and speltoid spike characteristics of the plants overexpressing miR172 are particularly suggestive of a limited role of the V329I polymorphism because the residual *Q-5A* transcript (39-45%, Fig. 3B) is expected to encode a protein carrying the V329I polymorphism.

Using dual*-*luciferase sensors, we demonstrated that the silent C-to-T transition in the *Q-5A* miR172 target site is sufficient to reduce the efficiency of miR172 cleavage (Fig. 1D,E). However, miR172 retained some residual activity on the *Q* target site sensor compared with a resistant target site that includes five additional mismatches (Fig. 1D,E). This result predicts that additional mutations in the miR172 site of *Q-5A* should reduce the silencing efficiency of miR172 even further and generate spikes that are more compact. Interestingly, the compact mutant MCK2617, isolated in hexaploid wheat, has a second mutation in the *Q-5A* miR172 target site [GenBank JX524755.1 (Kosuge et al., 2012; Sormacheva et al., 2015)]. The authors hypothesized that this phenotype was the result of a separate mutation in a locus close to the *Q* gene, but we suggest that the compact spike phenotype in this mutant is caused by the second mutation in the miR172 target site.

Results from our transgenic lines with reduced or increased miR172 levels and from the loss-of-function *Q-5A* mutants demonstrate that the balance between miR172 and *Q* gene expression is crucial for normal spike development. Alterations of this balance are likely to be responsible for the differences previously observed among lines carrying different dosages of *Q* and *q* alleles. The increasingly more compact spikes in trisomic and tetrasomic 5A lines (increasing dosage of *Q-5A*) (Muramatsu, 1963) can be explained by an increase in the proportion of intact *Q-5A* transcripts that escape a limiting amount of miR172. Competition for a limiting amount of miR172 can also explain the subcompact head spikes observed in plants carrying five or six doses of the *q-5A* allele (Muramatsu, 1963). The residual effect of the *q-5B* pseudogene on threshability and spike compactness (Zhang et al., 2011) can be explained by the alteration of the relative cellular concentration of miR172 and the total target pool (miRNA:target ratio) (Bosson et al., 2014). The *q-5B* pseudogene encodes a truncated protein but remains transcriptionally active and carries a functional miR172 binding site (Zhang et al., 2011). It would be interesting to examine the sequence and expression of *miR172* loci and the variation in total miR172 target pools in wheat germplasm with altered spike morphology.

In summary, our study shows that large changes in *AP2-5* levels can alter the identity of glumes and lemmas, generate supernumerary florets, and sterile basal spikelets, with potential negative impacts on grain yield or shattering. However, the more subtle increases in *AP2-5* generated by the mutation in its miR172 target site were sufficient to affect grain threshability without triggering detrimental homeotic changes.

## MATERIALS AND METHODS

### Plant materials and growth conditions

The tetraploid wheat variety Kronos (*Triticum turgidum* cv. *Kronos*) used in this study has the *Q*-*5A* allele, which confers the subcompact spike phenotype and free-threshing character. The mutants for *Q-5A* were identified in the sequenced TILLING populations of Kronos (Krasileva et al., 2017). The three selected truncation mutations were confirmed in M_4_ grain using the genome-specific primers described in Table S1.

Transgenic plants were grown in PGR15 growth chambers (Conviron) adjusted to 16 h of light (22°C) and 8 h of darkness (18°C). Intensity of the sodium halide lights measured at plant head height was ∼260 µM m^−2^ s^−1^. The M_4_ TILLING plants, the F_2_ population derived from the cross between Kronos and the K3946 mutant, and the F_1_ plants derived from the cross between *T. turgidum* subsp. *dicoccon* CItr 14454 (*T. dic.* 454) and MIM172#4 were grown in the greenhouse with average day and night time temperatures of 25°C and 17°C, respectively.

### Vectors

To overexpress miR172, we PCR amplified a 164 bp fragment from the *miR172d-6AL* locus that includes the complete miR172d precursor (premiR172d) from Kronos genomic DNA (primers listed in Table S1). The premiR172d sequence was cloned into the binary vector pLC41 (Japan Tobacco, Tokyo, Japan) downstream of the maize *UBIQUITIN* promoter. To generate the artificial MIM172, we synthesized the wheat ortholog of the natural miR399 target mimic *IPS1* gene described in *Arabidopsis* (Franco-Zorrilla et al., 2007) and replaced the sequence complementary to miR399 by a sequence complementary to miR172. The complete sequence of MIM172 is provided in Fig. S13. The MIM172 construct was cloned into pLC41 downstream of the maize *UBIQUITIN* promoter.

To generate the miR172 target site sensors, we obtained the dual-luciferase reporter plasmid (ORF sensor) (Liu and Axtell, 2015) from Addgene (#55207). The target sites were generated by hybridizing synthetic oligonucleotides flanked by *Avr*II and *Age*I sites and ligating these into the ORF-Luc previously digested with the same enzymes, with confirmation by sequencing (primers listed in Table S1). For the *N. benthamiana* experiments, the premiR172d sequence was cloned downstream of a 35S promoter in the Gateway binary vector pGWB14 (Nakagawa et al., 2007).

Kronos embryos were transformed with the pLC41 constructs using *Agrobacterium* and selected as described in the supplementary Materials and Methods.

### *Agrobacterium tumefaciens*-mediated transient expression in *N. benthamiana*

The dual-luciferase experiments were performed as described previously (Liu and Axtell, 2015). The same leaf samples collected for RNA extraction were used for the dual-luciferase activity. Luciferase activity was measured on a TriStar LB 941 Multimode Microplate Reader (Berthold Technologies).

### Morphological traits

Threshability and anatomical changes in glumes were assessed as described in the supplementary Materials and Methods.

### *In situ* mRNA hybridization analysis

*In situ* hybridization for *AP2-5* was conducted using an antisense probe generated from Kronos spike RNA as described in the supplementary Materials and Methods.

### qRT-PCR analysis

RNA samples were extracted from flag leaves (Ubi::miR172 experiment) and spikes (MIM172 experiment, TILLING mutants) using the Spectrum Plant Total RNA Kit (Sigma-Aldrich); we followed Protocol A, which allows purification of total RNA including small RNA molecules.

For the spike section experiment, samples were collected and dissected in RNA*later* (Ambion). Each biological replicate consisted of seven spikes. RNA was extracted from the section samples using TRIzol (Invitrogen) following the manufacturer's instructions, except in the RNA precipitation step where the aqueous phase and isopropanol were mixed at a 1:1 ratio and incubated for 2 h at −20°C. Total RNA was treated with RQ1 RNase-free DNase (Promega). cDNA synthesis was carried out using SuperScript II reverse transcriptase (Invitrogen). mRNA and miRNAs were reverse transcribed together starting from 1 µg total RNA. We used the oligo(dT) primer for mRNA. Mature miR172 levels were determined by stem-loop RT-qPCR as described previously (Chen et al., 2005). The reverse primer for small nucleolar RNA 101 (snoR101), which is the reference used to normalize miRNA in the qRT-PCR, was also included in the reverse transcription. qPCR was performed using SYBR Green and a 7500 Fast Real-Time PCR system (Applied Biosystems). *ACTIN* was used as an endogenous control for mRNAs. Primers are listed in Table S1.
